# A Multidrug ABC Transporter with a Taste for Salt

**DOI:** 10.1371/journal.pone.0006137

**Published:** 2009-07-10

**Authors:** Saroj Velamakanni, Calvin H. F. Lau, Daniel A. P. Gutmann, Henrietta Venter, Nelson P. Barrera, Markus A. Seeger, Barbara Woebking, Dijana Matak-Vinkovic, Lekshmy Balakrishnan, Yao Yao, Edmond C. Y. U, Richard A. Shilling, Carol V. Robinson, Peter Thorn, Hendrik W. van Veen

**Affiliations:** 1 Department of Pharmacology, University of Cambridge, Cambridge, United Kingdom; 2 Department of Chemistry, University of Cambridge, Cambridge, United Kingdom; University of Helsinki, Finland

## Abstract

**Background:**

LmrA is a multidrug ATP-binding cassette (ABC) transporter from *Lactococcus lactis* with no known physiological substrate, which can transport a wide range of chemotherapeutic agents and toxins from the cell. The protein can functionally replace the human homologue ABCB1 (also termed multidrug resistance P-glycoprotein MDR1) in lung fibroblast cells. Even though LmrA mediates ATP-dependent transport, it can use the proton-motive force to transport substrates, such as ethidium bromide, across the membrane by a reversible, H^+^-dependent, secondary-active transport reaction. The mechanism and physiological context of this reaction are not known.

**Methodology/Principal Findings:**

We examined ion transport by LmrA in electrophysiological experiments and in transport studies using radioactive ions and fluorescent ion-selective probes. Here we show that LmrA itself can transport NaCl by a similar secondary-active mechanism as observed for ethidium bromide, by mediating apparent H^+^-Na^+^-Cl^−^ symport. Remarkably, LmrA activity significantly enhances survival of high-salt adapted lactococcal cells during ionic downshift.

**Conclusions/Significance:**

The observations on H^+^-Na^+^-Cl^−^ co-transport substantiate earlier suggestions of H^+^-coupled transport by LmrA, and indicate a novel link between the activity of LmrA and salt stress. Our findings demonstrate the relevance of investigations into the bioenergetics of substrate translocation by ABC transporters for our understanding of fundamental mechanisms in this superfamily. This study represents the first use of electrophysiological techniques to analyze substrate transport by a purified multidrug transporter.

## Introduction

In order to evade the toxic effects of noxious compounds, prokaryotic and eukaryotic cells have developed several resistance mechanisms. One of these mechanisms involves the active extrusion of structurally-unrelated compounds from the cell by multidrug transporters [1]. The expression of these systems is a major cause of reduced efficacy of agricultural fungicides and herbicides [2], [3], and failure of chemotherapeutic treatment of tumours [4]–[6] and infections by pathogenic microorganisms [7]. Since the discovery of the human multidrug transporter ABCB1 (also termed multidrug resistance P-glycoprotein MDR1) [8], [9], homologues of ABCB1 have been identified in a variety of microorganisms. Among these, one of the most extensively studied proteins is LmrA from the non-pathogenic bacterium *Lactococcus lactis* [10].

LmrA is a half-transporter that contains an N-terminal membrane domain (MD) followed by a nucleotide-binding domain (NBD). By analogy to ABCB1 in which two half-transporters are fused into a single polypeptide, LmrA homodimerises to form the transport-active complex [11]. LmrA can functionally substitute for ABCB1 in human lung fibroblasts, and exhibits a substrate specificity similar to that of the human protein [12]. Its specificity is also similar to that of bacterial homologues such as HorA in *Lactobacillus brevis*, which is associated with resistance to iso-α-acids (in hopped beer) at increased copy number of the *horA*-containing plasmid pRH45 [13], [14], and the recently crystallized, genomically-encoded MsbA from *Escherichia coli*, *Salmonella typhimurium*, and *Vibrio cholera* [15]–[19] and Sav1866 from *Staphylococcus aureus* [20], [21]. Studies on the pharmacological properties of multidrug ABC transporters (reviewed in [1], [19], [22], [23]) revealed that the ability of these systems to transport multiple drugs is based on direct drug-protein interactions. A recent crystal structure of mouse ABCB1a demonstrates substrate binding at the top of an internal cavity at the dimer interface between the two half-transporters near the leaflet-leaflet interface of the membrane [24], consistent with biochemical observations on MsbA [19].

Gram-positive lactic acid bacteria play an important role in the food industry due to their fermentative capacities [25]. *L. lactis* is the main component of the starter cultures used for cheese making. Its main activity during milk fermentation is the conversion of lactose to lactic acid, which results in the lowering of the pH in the product to prevent the growth of spoilage and pathogenic microorganisms [26]. During this process as well as in its natural habitats, *L. lactis* is subjected to stressful conditions which, in addition to acidity, include exposure to antibacterial peptides (e.g. bacteriocins), toxic metabolic products (e.g. bile salts and fatty acids), secondary metabolites (e.g. antibiotics), and high osmolarity/dehydration; NaCl is an important constituent of cheese which can reach concentrations of up to 0.5 M.

In this paper, we report that LmrA can mediate a H^+^-Na^+^-Cl^−^ symport reaction and can enhance survival of salt-adapted cells during downshifts in the extracellular ion concentration. Observations on the redundancy and overlapping drug specificities of multidrug ABC transporters [27] and the examples of regulation of their expression independent of multiple drugs [28] have indicated that some of these ABC transporters might have alternative or additional physiological roles. Our findings on LmrA now provide an interesting example of this notion.

## Results

### Purified LmrA-MD and LmrA do not contain additional binding partners

**Figure 1:**
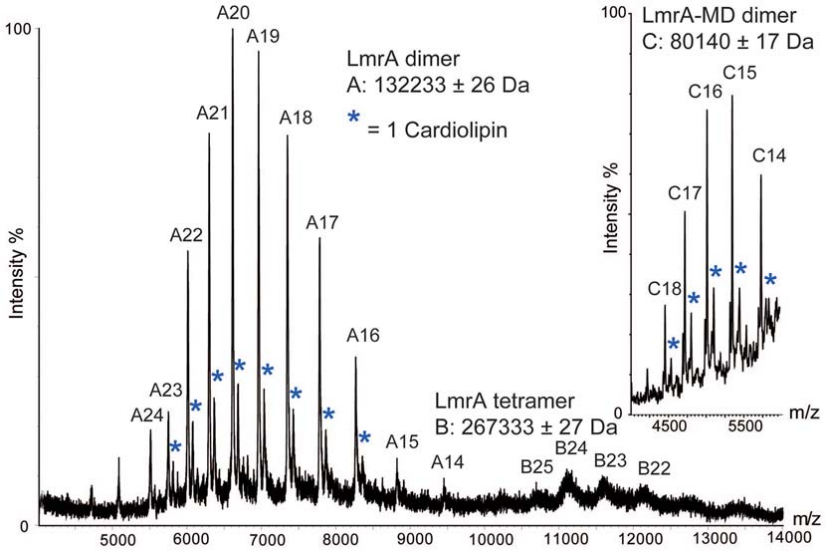
Mass spectra of purified LmrA and LmrA-MD showing predominant homodimer formation for both proteins. Peaks assigned to binding of one cardiolipin molecule are labelled (blue stars), and measured molecular masses are shown.

In previous work in *L. lactis*, we engineered a truncated LmrA protein, termed LmrA-MD, which lacks the NBD [29]. Studies on the purified protein in proteoliposomes revealed that homodimeric LmrA-MD functions as a secondary-active multidrug translocator by catalyzing reversible ethidium-proton symport without a requirement for ATP [30]. This observation was recently extended to full-length LmrA in proteoliposomes [31]. As our observations on ion-coupled transport are the first reported for a purified multidrug ABC transporter, we used a recently described method involving nano-electrospray mass spectrometry [32] to confirm the subunit stoichiometry, lipid binding, and purity of our preparations of LmrA and LmrA-MD. Predominant well-resolved peaks corresponded to molecular masses of 132233±26 Da and 80140±17 Da and were consistent with homodimers of LmrA and LmrA-MD (Figure 1); calculated molecular masses of the dimeric complexes were 132312 and 80218 Da, respectively. Peaks corresponding to tetramers of LmrA and LmrA-MD were also observed in both spectra and are likely due to a low level of association of dimers within the micelles. Lipid binding was also evident for both proteins with masses consistent with a cardiolipin (1352 Da). Notably our protein preparations did not contain additional (membrane) proteins that could act as binding partners of LmrA or LmrA-MD.

### Ionic currents in LmrA-MD-containing proteoliposomes

**Figure 2:**
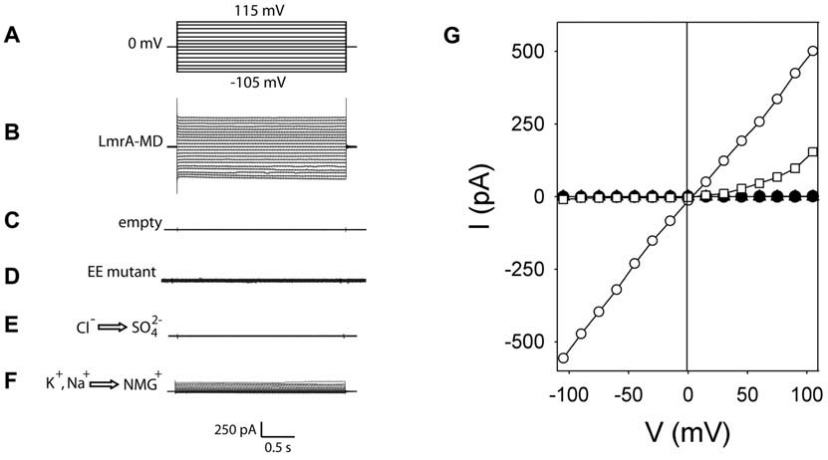
Electrophysiological analyses of ion transport in proteoliposomes by ‘dip-tip’ method. A, Voltage step protocol from a holding potential of 0 mV to various voltages (ranging from −105 mV to +115 mV), back to 0 mV. B, C, D, Current traces for LmrA-MD (B) or empty liposomes without protein (C) or EE LmrA-MD (D) in the presence of 10 mM NaCl. E, F, Current traces for LmrA-MD in the presence of SO_4_^2−^ instead of Cl^−^ (E), or NMG^+^ instead of Na^+^ and K^+^ (F). G, I–V curves from the traces in (B–F) (○, LmrA-MD; •, replacement of Cl^−^ by SO_4_^2−^; □, replacement of K^+^/Na^+^ by NMG^+^; ▿, EE; ▵, empty liposomes; the latter two traces are hidden behind •). (*n* = 15)

We studied the mechanism of ion transport by LmrA-MD in more detail. Surprisingly, when proteoliposomes containing purified and functionally reconstituted LmrA-MD were fused with a phospholipid bilayer in the tip of a glass pipette, macroscopic currents associated with thousands of LmrA-MD molecules could be recorded in response to hyper- or depolarizing pulses from a holding potential of 0 mV (Figure 2A). The current changed linearly with voltage and showed no evidence for rectification, indicating comparable rates of ion conductance in uptake and efflux directions (Figure 2B). No significant currents could be recorded for empty control liposomes (Figure 2C) or proteoliposomes containing a mutant LmrA-MD protein in which two basic residues (K32 and R34) in the cytosolic N-terminal elbow helix are replaced by E (termed EE LmrA-MD) (Figure 2D). This mutant was equally well expressed in cells as wildtype. In addition, the mutation did not alter ethidium and Hoechst 33342 binding by purified LmrA-MD or LmrA in detergent solution, or the basal ATPase activity of the latter, suggesting that no gross conformational changes were introduced (data not shown). Currents were also absent upon the replacement of Cl^−^ in the internal and external bath solutions by SO_4_^2−^, and were strongly reduced by the replacement of K^+^ and Na^+^ by N-methyl-D-glucosamine^+^ (NMG^+^) (Figure 2E–G), demonstrating that the currents are a result of LmrA-MD mediated Cl^−^ and Na^+^ and/or K^+^ transport.

### Proton-coupled chloride transport

**Figure 3 pone-0006137-g003:**
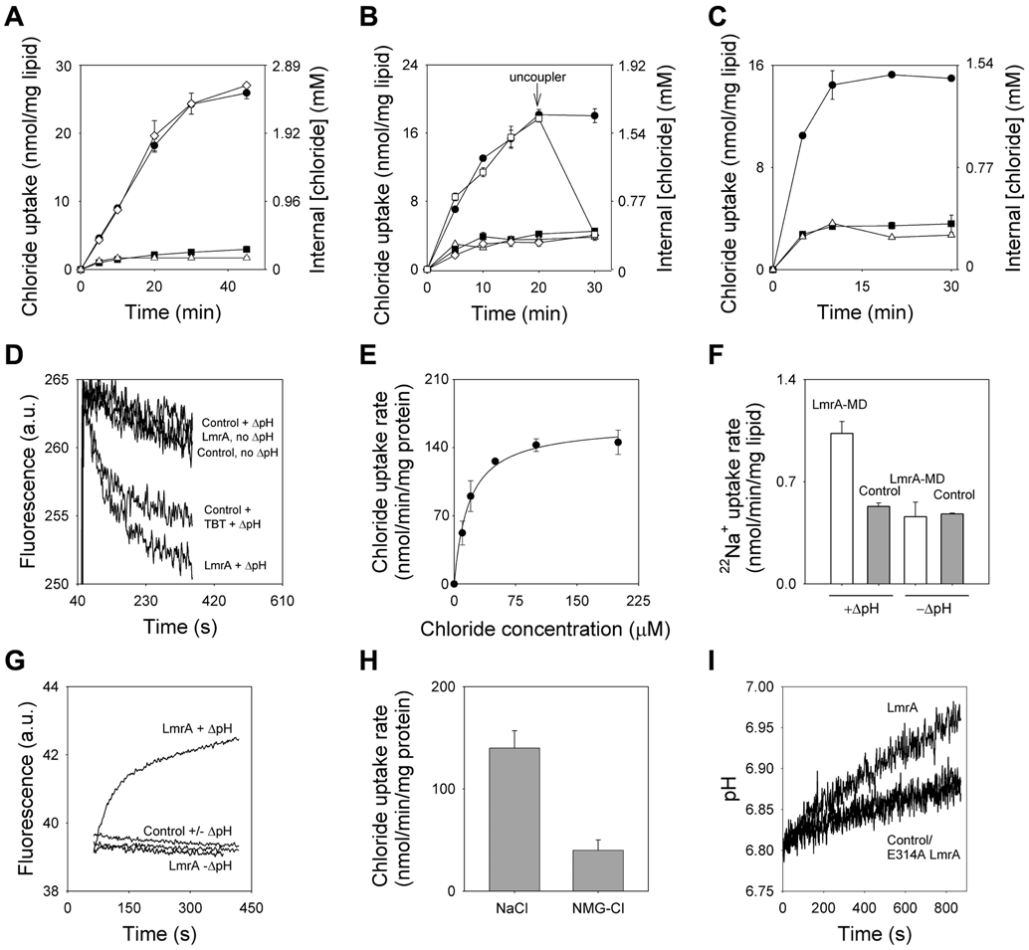
Ion-coupled transport in proteoliposomes. A, B, ^36^Cl^−^ uptake (100 µM) by LmrA-MD (•), E314A LmrA-MD (⋄), EE LmrA-MD (▪) or empty liposomes (▵) in the presence of a Δψ (interior negative) of −120 mV (A) or -ZΔpH (interior alkaline) of −49 mV (B). In the duplicate experiment for LmrA-MD (□) in (B), the addition of uncoupler (valinomycin plus nigericin, 1 µM each) at the arrow resulted in efflux of accumulated ^36^Cl^−^, indicating concentrative uptake of the ion. C, ΔpH (interior alkaline)-dependent ^36^Cl^−^ uptake by LmrA (•), EE LmrA (▪) or empty liposomes (▵). D, ΔpH (interior alkaline)-dependent uptake of non-radioactive Cl^−^ (1 mM) by LmrA is observed as a quench in the fluorescence of the SPQ fluorophore trapped in the lumen of the proteoliposomes. Quenching was also observed in empty liposomes (control) in the presence of the Cl^−^/OH^−^ antiporter TBT-Cl (1 µM). E, Kinetic analysis of ΔpH (interior alkaline)-dependent ^36^Cl^−^ uptake by LmrA. F, ΔpH (interior alkaline)-dependent uptake of ^22^Na (25 µM) by LmrA-MD. G, Uptake of unlabelled Na^+^ (10 mM) by LmrA was detected as an increase in the fluorescence of the membrane-impermeable sodium green probe trapped in the lumen. H, Na^+^ (100 µM) stimulates the ΔpH-dependent uptake of ^36^Cl^−^ (100 µM) by LmrA compared to control containing 99 µM NMG^+^ plus 1 µM Na^+^. I, H^+^ efflux in proteoliposomes loaded with pH probe BCECF in the presence of an outwardly directed NaCl gradient. Control, empty liposomes. (*n* = 5)

The transport of Cl^−^ in LmrA-MD-containing proteoliposomes was further studied in transport assays using radioactive ^36^Cl^−^. Upon the imposition of a transmembrane potential (Δψ) of −120 mV (inside negative), proteoliposomes accumulated ^36^Cl^−^ about 17.4-fold above the equilibration level obtained for empty liposomes ([Cl^−^]_in_ = 2.79±0.07 mM for LmrA-MD versus 0.16±0.04 mM for control) (Figure 3A). In contrast, ^36^Cl^−^ transport by the EE LmrA-MD was strongly reduced and almost identical to levels in empty liposomes. The accumulation of Cl^−^ by wildtype LmrA-MD at the negative side of the proteoliposomal membrane points to the electrogenic co-transport of Cl^−^ and cations.

One type of cation that is co-transported with Cl^−^ is H^+^. The imposition of a transmembrane chemical proton gradient (-ZΔpH, interior alkaline) of −49 mV in proteoliposomes by the outward diffusion on acetic acid (pH 7.6_in_/pH 6.8_out_) [29] (Figure 3B) was accompanied by a 4.2-fold accumulation of ^36^Cl^−^ above the equilibration level in empty liposomes ([Cl^−^]_in_ = 1.61±0.12 mM for LmrA-MD versus 0.38±0.08 mM for control). Uptake by EE LmrA-MD was comparable to control. The addition of an uncoupler that dissipated the imposed ΔpH initiated efflux of previously accumulated ^36^Cl^−^, providing evidence for concentrative ion uptake by LmrA-MD (Figure 3B). The uphill movement of Cl^−^ against a concentration gradient at the expense of H^+^ moving downhill is consistent with the notion that LmrA-MD act as a transporter. The uptake of ^36^Cl^−^ was also observed when a ΔpH (interior alkaline) was imposed by pH jump (pH 7.6_in_/pH 6.8_out_), but not upon imposition of a reversed ΔpH (pH 6.8_in_/pH 7.6_out_) by this method (data not shown). Previous findings on ethidium-proton co-transport by LmrA-MD and LmrA indicated that the E314A substitution in transmembrane helix 6 abrogates ΔpH-dependence but not Δψ-dependence of this reaction [29], [30]. In agreement with this, the E314A mutation strongly inhibited the ΔpH-dependent transport of ^36^Cl^−^, whereas the Δψ-mediated transport was unaffected (Figure 3A–B).

The observations for LmrA-MD were also relevant for full-length LmrA, as ΔpH (pH 7.6_in_/pH 6.8_out_)-driven uptake of ^36^Cl^−^ resulted in a 4.4-fold accumulation of the ion in proteoliposomes containing LmrA compared to empty liposomes ([Cl^−^]_in_ = 1.54±0.20 mM for LmrA versus 0.35±0.08 mM for control) (Figure 3C). The ΔpH (interior alkaline)-dependent uptake of Cl^−^ could also be detected when a Cl^−^-sensitive fluorophore was trapped in the liposomal lumen (Figure 3D). The accumulation of Cl^−^ was observed as a fluorescence quenching with an initial rate of (46.2±0.9)×10^−3^ a.u./s in LmrA-containing proteoliposomes versus (13.2±7.6)×10^−3^ a.u./s in empty liposomes. Upon the addition of the Cl^−^/OH^−^ ion exchanger tributylin chloride (TBT-Cl) to the liposomes in the presence of the ΔpH, a similar quenching of fluorescence was obtained as that associated with LmrA activity (Figure 3D), supporting the notion that LmrA mediates chloride-proton co-transport. Measurement of the rate of ΔpH-dependent ^36^Cl^−^ transport by LmrA as a function of the Cl^−^ concentration indicated a high apparent affinity (Michaelis constant, K_m_) of 17.9±3.7 µM Cl^−^ and a maximum transport rate (V_max_) of about 142 nmol/min/mg protein (Figure 3E). This V_max_ is comparable to the maximum ATPase activity of purified LmrA (about 150 nmol/min/mg protein) obtained at saturating concentrations of Mg-ATP (above 6 mM) [31]. The rates of Cl^−^ transport obtained for the EE and E314A mutants were too low to allow a reliable determination of kinetic parameters.

At steady state in Figure 2A–C, the outwardly directed chemical Cl^−^ gradient is in equilibrium with the inwardly directed Δψ and ΔpH (*Z*log[Cl^−^]_in_/[Cl^−^]_out_ = −(*n*Δψ−*m*ZΔpH) in which *Z* = 59 mV). From the data, *n* was calculated as 0.7±0.1 (Figure 3A), and *m* = 0.8±0.1 (Figure 3B) and 0.8±0.2 (Figure 3C). These estimates indicate an apparent net stoichiometry of 2(+)/Cl^−^ anion and 1 H^+^/Cl^−^, respectively, pointing to electrogenic symport of 1X^+^∶1Cl^−^∶1H^+^.

### Coupled transport of NaCl and protons

Consistent with the electrophysiology experiments showing Na^+^/K^+^ conductance by LmrA-MD, Na^+^ can participate in the X^+^-Cl^−^-H^+^ co-transport reaction. Upon the imposition of a ΔpH (pH 7.6_in_/pH 6.8_out_), ^22^Na^+^ uptake in proteoliposomes containing LmrA-MD was observed above the equilibration level in empty liposomes consistent with sodium-proton co-transport (Figure 3F). The ΔpH-dependent accumulation of Na^+^ could also be monitored in LmrA-containing proteoliposomes loaded with a Na^+^-sensitive fluorophore (Figure 3G). In both types of experiments, LmrA(-MD)-mediated Na^+^ transport was strongly reduced in the absence of the ΔpH and identical to levels in empty liposomes. In agreement with H^+^-Na^+^-Cl^−^ co-transport, the presence of Na^+^ stimulated the ΔpH-dependent uptake of ^36^Cl^−^ in LmrA-containing proteoliposomes compared to a control in which the Na^+^ concentration was reduced 100-fold by replacement with NMG^+^ (Figure 3H). In analogous experiments with the radioactive K^+^ analog ^86^Rb^+^, no ΔpH-dependent accumulation was observed in LmrA-MD containing proteoliposomes above control (data not shown), which most likely reflects the low affinity of LmrA-MD for K^+^ relative to Na^+^.

As the ΔpH can drive Na^+^ and Cl^−^ transport by LmrA, downhill transport of these ions by LmrA might be able to regenerate this ΔpH. To test this hypothesis, (proteo)liposomes were prepared containing a pH-sensitive fluorophore. When the (proteo)liposomes were diluted to create an outwardly directed NaCl concentration gradient (5 mM_in_/0.05 mM_out_) the simultaneous efflux of Na^+^, Cl^−^ and protons by LmrA was observed as an increase in luminal pH of (15.1±1.1)×10^−3^ units/min (Figure 3I). The enhanced proton efflux was not observed for empty control liposomes or proteoliposomes containing E314A LmrA (pH increase of (5.2±1.0)×10^−3^ versus (6.2±1.3)×10^−3^ units/min, respectively). No differences in the luminal pH were observed upon dilution without the imposition of the NaCl gradient (data not shown).

### Ion transport in intact cells

**Figure 4 pone-0006137-g004:**
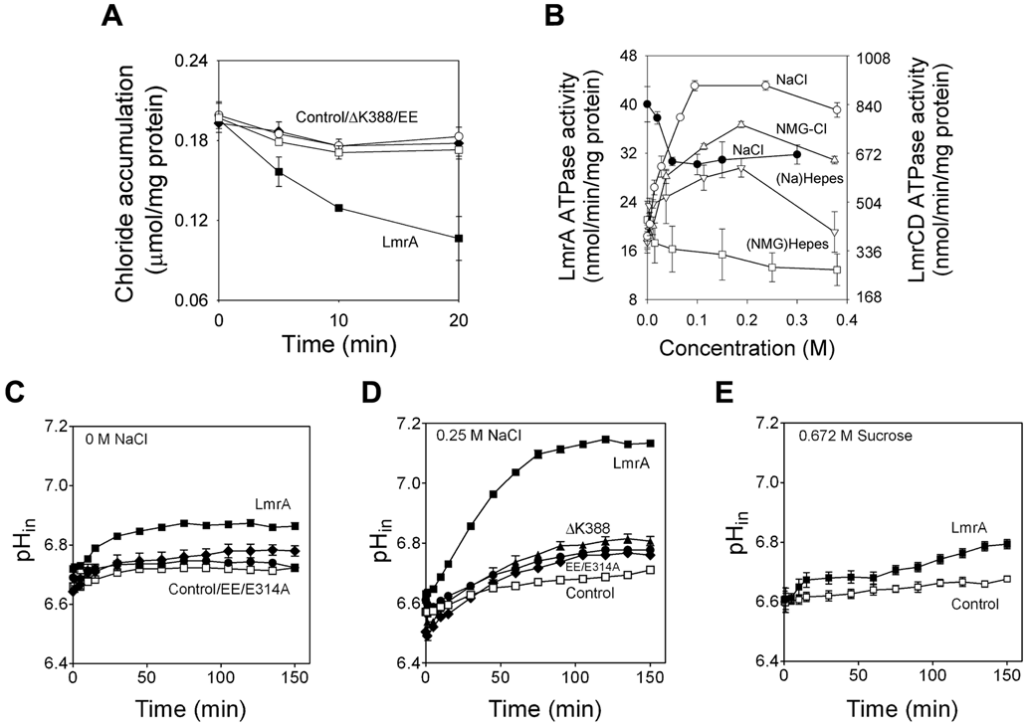
Ion transport in intact cells. A, Cl^−^ efflux in cells preloaded with Na^36^Cl (100 µM) upon the addition of glucose (▪, LmrA; □, non-expressing control; ⧫, EE LmrA; ○, ΔK388 LmrA). B, Effect of the concentration of Na^+^, NMG^+^ or Cl^−^ on the ATPase activity of purified LmrA (open symbols) or LmrCD (•) measured at 2 mM Mg-ATP. C,D,E, H^+^ efflux in energized cells, loaded with pH probe CFDASE to monitor the intracellular pH (pH_in_) in the absence (C) or presence of (D) 0.25 M NaCl or (E) 0.672 M sucrose in the external buffer (each equivalent to 521 mOsm) (▪, LmrA; □, non-expressing control; ⧫, EE LmrA; •, E314A LmrA; ▴, ΔK388 LmrA). Metabolic energy was generated in the cells by the addition of 20 mM glucose (at t = 0 min in the figures), 15 min after the addition of the NaCl or sucrose or solvent control. (*n* = 8)

The addition of glucose as a source of metabolic energy to cells pre-equilibrated with 100 µM Na^36^Cl elicited ^36^Cl^−^ efflux by LmrA but not by an ATPase-deficient LmrA mutant in which the catalytic K388 residue in the Walker A region of the NBD was deleted (ΔK388) [31] (Figure 4A). Efflux was also not observed for non-expressing control cells or cells expressing EE LmrA. The notion that LmrA can transport salt in an ATP-dependent fashion is supported by the inorganic ion-dependent stimulation of the basal ATPase activity of the protein in detergent solution (Figure 4B). A maximal stimulation was observed for NaCl, whereas an intermediate level of stimulation was obtained for (Na)Hepes and (NMG)Cl. On the other hand, (NMG)Hepes did not affect the basal ATPase activity suggesting that the ion-dependent stimulation of the LmrA-ATPase activity is due to a specific interaction with Na^+^ and Cl^−^. This notion is also supported by the observation that, in contrast to LmrA, the ATPase activity of the purified multidrug ABC transporter LmrCD from *L. lactis* [33] is not stimulated by NaCl up to a concentration of 0.3 M (Figure 4B).

H^+^-Na^+^-Cl^−^ co-transport by LmrA was also indicated in H^+^ efflux measurement from cells. Estimation of the intracellular pH in the cells with a fluorescent pH indicator revealed an alkalinization of the cytosol in LmrA expressing cells, consistent with H^+^ efflux by LmrA (Figure 4C). The external pH was constant during the time course of these experiments (not shown). Interestingly, the addition of 0.25 M NaCl to the extracellular buffer did not have an immediate effect on alkalinization, but strongly stimulated proton efflux after a prolonged exposure of the cells for 15 min (Figure 4D). This time dependence suggests that the H^+^-Na^+^-Cl^−^ co-transport reaction is enhanced by elevated concentrations of NaCl in the cytosol. NaCl could, to a limited extend, be replaced by KCl at a similar osmolality (521 mOsm) (not shown) but could not be replaced by sucrose (Figure 4E), indicating an ionic requirement in the stimulation. The E314A, EE and ΔK388 mutations in LmrA strongly reduced the elevation of the cytosolic pH in the presence of NaCl (Figure 4D), consistent with their inhibition of LmrA-mediated ion transport (Figure 3–4A).

### LmrA activity enhances cell survival

**Figure 5:**
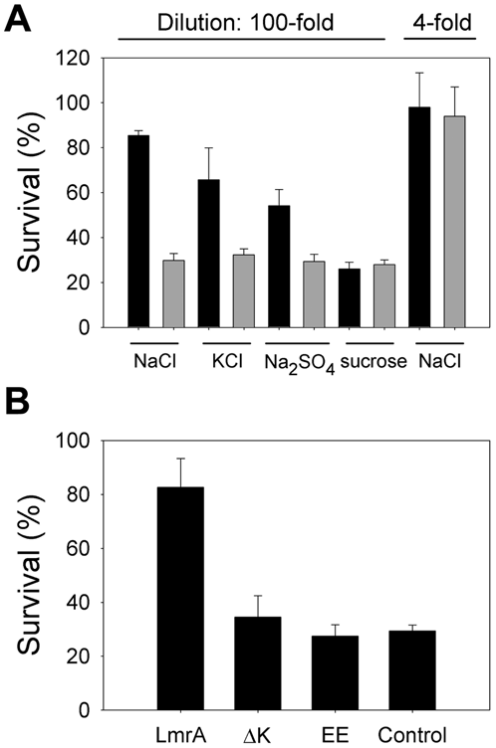
LmrA activity enhances cell survival. A, Viability of energized cells (solid bar, LmrA; grey bar, non-expressing control) adapted for 30 min in buffer containing 0.5 M sucrose without or with 100 mM NaCl, 100 mM KCl or 50 mM Na_2_SO_4_, followed by 100-fold dilution into ultrapure water, or in buffer containing 0.125 M sucrose and 25 mM NaCl (control referred to as 4-fold dilution). B, Effect of mutations in LmrA on the viability after dilution of cells pre-exposed to 100 mM NaCl plus 0.5 M sucrose under conditions as in (A). (*n* = 10)

A relationship was found between LmrA activity and cell viability during ionic downshift. When cells were pre-exposed to 100 mM NaCl plus 0.5 M sucrose for 30 min, and subsequently diluted 100-fold in ultra pure H_2_O, the cells expressing LmrA survived the downshock with a significant gain of viability compared to the non-expressing control (Figure 5A). Cell survival required exposure to NaCl, which could partially be substituted by KCl and Na_2_SO_4_, but not by sucrose only (Figure 5A). These data are consistent with the stimulatory effect of these salts, but not of sucrose, on LmrA-mediated H^+^ efflux (Figure 4D). In contrast, no differences in cell survival between LmrA expressing cells and control cells were observed following a minor 4-fold downshock (Figure 5A). The survival of NaCl adapted cells was strongly reduced for the EE and ΔK388 mutants (Figure 5B), which are impaired in ion transport (Figure 2–4), indicating that LmrA itself can diminish irreversible damage in salt-exposed cells during the ionic downshock.

## Discussion

Collectively, the observations on proton-coupled Cl^−^ transport, proton-coupled Na^+^ transport, Na^+^-dependent Cl^−^ transport, and NaCl-coupled proton transport by purified protein in proteoliposomes (Figure 3) provide compelling evidence that both LmrA-MD and LmrA can mediate an electrogenic H^+^-Na^+^-Cl^−^ symport reaction. An alternative explanation in which LmrA-MD/LmrA might regulate the activity of an unidentified, associated membrane protein appears less likely since an associated protein was not observed in our purified protein preparations in analyses based on silver–stained SDS-PAAGE, N-terminal amino acid sequencing (data not shown) and non-dissociating mass spectrometry (Figure 1). Consistent with previous cross-linking experiments on LmrA and LmrA-MD [11], [29], and studies demonstrating that (i) an inactive LmrA mutant exhibits negative dominance over an active wild-type LmrA protein in a co-reconstituted liposomal system, and (ii) the covalent fusion of two wild-type LmrA monomers yields a functional transporter whereas the fusion of a wild-type monomer and the inactive mutant monomer does not [11], the mass spectra (Figure 1) showed predominant peaks for the LmrA and LmrA-MD dimers, and showed no evidence for other proteinacious binding partners. Ion transport was strongly inhibited by the distinct EE and E314A mutations in the MD, and Walker A ΔK388 mutation in the NBD (Figure 2–4) the latter of which significantly reduces the LmrA ATPase activity down to 4% of wildtype level [31], [34]. Taken together, the data suggest that LmrA/LmrA-MD itself is responsible for inorganic ion transport.

Current advancements in the biochemistry and structural biology of ABC transporters indicate that these systems operate by a mechanism in which binding sites in the MDs for transport substrate(s) are alternately exposed to the inside and outside surface of the membrane in response to the ATP-driven dimerization of NBDs and their dissociation following ATP hydrolysis (for reviews, see Ref. 35, 36). The strong inhibition by the ΔK388 mutation of LmrA-mediated Cl^−^ and H^+^ efflux from ATP-containing cells (Fig. 4A–D) is consistent with this notion, and suggests that substrate (H^+^, Na^+^ and Cl^−^) efflux is dependent on phosphate-bond energy derived from ATP hydrolysis. Evidence for ATP-dependence of transport by LmrA was also previously obtained in drug transport studies [10], [11], [31], [37]. However, electrochemical ion gradient-dependent H^+^-Na^+^-Cl^−^ symport by LmrA in proteoliposomes in the absence of ATP implies that this reaction can occur in the absence of a catalytic cycle at the NBDs; indeed, the same reaction can be detected for LmrA-MD that does not contain the NBD. Hence, in the absence of the successful dimerization of NBDs and/or their conformational coupling in the LmrA transporter, the MDs operate in a reversible, secondary-active mode in which the direction of transport is dictated by the orientation and magnitude of the electrochemical substrate (H^+^, Na^+^ and Cl^−^) gradients. It is interesting to note that for the LmrA homologue Sav1866, which exhibits a similar reversibility of transport as LmrA [21], the homodimer was co-crystallized with a Na^+^ ion bound in the hinge region connecting the α-helical and β-sheet subdomains in each NBD [20], where these ions might affect the dimerisation of NBDs and/or their conformational coupling to MDs. Ion sensing in LmrA might also occur by the cytosolic N-terminal elbow helix which is predicted to be in close proximity to the inside surface of the membrane [20]. In LmrA, this helix contains 8 basic residues (R10, K14, K18, K22, K26, R29, K32, R34) which could bind anions (Cl^−^) and which, in competition with cations (Na^+^), could interact with acidic lipids and/or neighbouring residues in transmembrane helix 2, 3 and 6. Consequently, the interactions at the elbow helix might occur in an ionic strength (NaCl)-dependent manner. The phenotype of the EE mutant (Figure 2–5) suggests that the simultaneous replacement of K32 and R34 by E in the elbow helix interferes with its function. Hence, ion-dependent regulatory mechanisms might exist in this type of multidrug ABC exporter that affect transport activity.

LmrA shares its ability to conduct Cl^−^ (Figure 2) with the only established ABC Cl^−^ channel CFTR [38], in which ATP binding triggers channel opening and ATP hydrolysis triggers channel closing through the effect of nucleotide binding/hydrolysis on NBD dimerisation [39]. In addition, LmrA might share its ability to conduct Cl^−^ with human ABCB1, the expression of which was reported to be associated with a Cl^−^ dependent regulation of intracellular pH in chinese hamster ovary fibroblasts [40], [41] and with volume-dependent Cl^−^ currents in mouse fibroblasts [42]. Whereas ABCB1 was suggested to act as a Cl^−^ channel [42], our data on LmrA-mediated, proton-motive force (interior negative and alkaline)-dependent accumulation of Cl^−^ in proteoliposomes (Figure 3A–D) and the release of pre-accumulated Cl^−^ from proteoliposomes upon dissipation of the proton-motive force by protonophore (Figure 3B) indicate that LmrA operates as a proton-coupled Cl^−^ transporter. Recently, members of the Chloride Channel (ClC) family were shown to mediate a rapid 2Cl^−^/H^+^ antiport reaction, indicating a close connection between transporters and channels in this protein family [43]–[46]. As LmrA and CFTR are structurally related proteins, our findings on LmrA-mediated Cl^−^ transport might point to a mechanistic relationship between transporters and channels in the ABC superfamily.

The observations on salt transport by LmrA and the increased survival of salt-adapted LmrA-expressing cells during ionic downshift (Figure 5) imply a novel link between LmrA activity and the homeostatic control of the intracellular pH and NaCl concentration, and represent an interesting example of the notion that multidrug ABC transporters can have physiological roles that go beyond the transport of toxic compounds. We found that LmrA expression does not confer resistance on lactococcal cells to NaCl or acidity during exponential growth (data not shown) where ion homeostasis is in place. Therefore, the beneficial action of LmrA on cell survival appears to be relevant during periods of disturbed ionic balance. It should be mentioned that the deletion mutant lacking the *lmrA* gene did not reveal a significant growth or survival phenotype compared to the wildtype background in *L. lactis* NZ9000 (data not shown) suggesting insufficient expression of genomic *lmrA* under laboratory conditions in M17 medium, or a redundancy in LmrA function as was observed for mechanosensitive channel proteins in *E. coli* [47], [48]. The MS data (Figure 1) indicate binding of a specific lipid, cardiolipin, by the LmrA dimer. As it was previously proposed that LmrA might act as a flippase for hydrophobic compounds including lipid [11], [12], [49], the potential role of lipid transport by LmrA in cell membrane homeostasis during salt stress (including the regulation of lipid composition, membrane tension and/or surface area) remains to be determined.

## Materials and Methods

### Cell and membrane preparations

The expression of LmrA, LmrA-MD, LmrA(-MD) mutants, and LmrCD in the drug-hypersensitive *L. lactis* strain NZ9000 Δ*lmrA* Δ*lmrCD* [31], [33], the solubilization and affinity-purification of these proteins in *n*-dodecyl *β*-D-maltoside (DDM, Anatrace)-containing solutions, and the reconstitution of purified proteins at a protein-lipid ratio of 1∶100 (w/w) in Triton X-100 (BDH Chemicals)-destabilized proteoliposomes (with an internal volume of 10.4 µl/mg lipid) in 50 mM KPi (pH 7.6), 2 mM MgSO_4_ was performed by methods as described previously [21], [29], [32], [49]. For mass spectrometry of LmrA, protein was purified as above using dodecyl octa(ethylene oxide) (C12E8, Anatrace) rather than DDM at equivalent w/v concentration. To determine the ATPase activity of LmrA, protein was solubilized in C12E8 and bound to Ni^2+^-nitrilotriacetic acid (Ni-NTA) resin as above, and subsequently washed and eluted with buffer containing 0.1% (w/v) fos-choline-12 (Anatrace).

### Genetic manipulations

Site directed mutagenesis was performed on pGHLmrA [30] using the QuikChange kit (Stratagene). The K32E/R34E (EE) double mutant was generated with primers 5′- GCA GAA CCA GAG TAT CTC TTT TTT GTG ATT G -3′ and 5′-TAC TCT GGT TCT GCT GCA CGA ATT AAT TTG-3′. To generate EE LmrA-MD, the coding region for the MD [29] was PCR amplified using the mutant *lmrA* gene as a template.

### Mass spectrometry

Analyses were performed using a nanoflow ES mass spectrometer Q-ToF2 (Micromass), which has been modified for transmission and detection of ions at high m/z ratios [50]. Aliquots of complex-containing solutions (2 µL at ∼10 µM concentration in 190 µM C12E8) were introduced via gold-coated nanoflow electrospray capillaries, prepared in-house. The following experimental parameters were used to record mass spectra of 1 mg/ml LmrA and LmrA-MD: needle voltage of 1.8 kV and MCP 2350 V, 150 V cone and 100 V collision voltages for LmrA-MD and 200 V both cone and collision voltages for LmrA.

### Electrophysiological measurements

Ion conductance was measured by the ‘dip-tip’ technique [51]. The external bath and the tip of a micropipette were filled with 10 mM (K)Hepes (pH 7.4), 1 mM MgCl_2_, 2 mM CaCl_2_, 1 mM KCl and 10 mM NaCl. A planar lipid bilayer in the tip of the pipette was formed using 1-palmitoyl-2-oleyl-*sn*-glycero-3-phosphocholine and 1,2-dioleoyl-*sn*-glycero-3-phosphoethanolamine (Avanti Polar lipids) in a ratio of 7∶3 (w/w), which increased the resistance of the pipette from about 7 MΩ to about 75 GΩ. Proteoliposomes prepared from synthetic lipids fused with the planar lipid bilayer following their addition to the external bath at a concentration of 19 µg protein/ml.

### Biochemical assays

The transport of radioactive chloride or sodium ions was measured by rapid filtration after 100-fold dilution in 50 mM KPi (pH 6.8), 2 mM MgSO_4_, or buffers and conditions as described [52], containing 100 µM Na^36^Cl (16 mCi/g) (MP Biomedicals) or 25 µM ^22^NaCl (100 mCi/mg) (Amersham Biosciences). The transport of H^+^ in proteoliposomes in 5 mM KPi (pH 7.6) or non-radioactive Cl^−^ (1 mM) or Na^+^ (10 mM) was measured by fluorimetry using 50 µM 2′,7′-bis-(2-carboxyethyl)-5-(and-6)-carboxyfluorescein (BCECF) [29], 300 µM 6-methoxy-N-(3-sulfopropyl) quinolinium (SPQ), or 1 mM membrane-impermeable sodium green (Molecular Probes), respectively, that was trapped in the lumen. Cells in phosphate-buffered saline were loaded with 30 μM 5(6)-carboxyfluorescein diacetate succinimidyl ester (CFDASE) (Molecular Probes) for detection of H^+^ transport [53], and washed and resuspended in 50 mM KPi (pH 6.0). Fluorescence was calibrated in the presence of nigericin (pH_in_ = pH_out_) using a micro pH electrode for measurement of pH_out_. ATPase activities on purified protein (1 µg in 30 µl buffer) were estimated by the malachite green method as described [31] using LmrCD in 0.05% (w/v) DDM or LmrA in 0.1% (w/v) fos-choline-12.

### Cell viability

Cell suspensions (OD_660_ = 0.5) were pre-incubated for 30 min in 50 mM (NMG)Hepes (pH 7.4) plus 20 mM glucose with additions as indicated in the legend to Figure 5A–B after which they were 100-fold diluted into ultra pure water. After 10 min, cells were diluted 10^6^-fold and 0.1 ml was plated onto M17 agar plates supplemented with 5 µg/ml chloramphenicol and 0.5% (w/v) glucose. The plates were incubated overnight at 30°C and the number of colony forming units (cfu)/plate was counted. The 100% value refers to cfu/plate immediately prior to the dilution of the cells in water, and was equal to 48 and 45 for LmrA-expressing and control cells, respectively.

### Data analysis

All statistical analyses were performed using the paired Student's *t*-test with a 95% confidence interval for the sample mean, based on *n* independent observations with different cell batches or (proteo)liposome preparations.
